# A Comparison of the Efficacy of Treatment With Fidaxomicin Versus Vancomycin in Clostridioides difficile Infection

**DOI:** 10.7759/cureus.48735

**Published:** 2023-11-13

**Authors:** Beatriz Diaz-Pollan, Sergio Carrasco Molina, Cristina Marcelo, Alejandro de Gea Grela, Patricia Martínez-Martín, María Jiménez-González, Francisco Moreno Ramos, Marta Mora-Rillo

**Affiliations:** 1 Internal Medicine Department, Infectious Diseases Unit, Hospital La Paz Institute for Health Research (IdiPAZ) Hospital Universitario La Paz, Madrid, ESP; 2 CIBERINFEC (Centro de Investigación Biomédica en Red de Enfermedades Infecciosas), Instituto de Salud Carlos III, Madrid, ESP; 3 Clinical Trials Unit (UCICEC), Hospital La Paz Institute for Health Research (IdiPAZ) Hospital Universitario La Paz, Madrid, ESP; 4 Pharmacy Department, Infectious Diseases Unit, Hospital La Paz Institute for Health Research (IdiPAZ) Hospital Universitario La Paz, Madrid, ESP

**Keywords:** recurrence, clostridioides difficile, cdi, vancomycin, fidaxomicin

## Abstract

Background

*Clostridioides difficile *infection (CDI) is a major cause of diarrhea in hospitalized adult patients. This study aims to evaluate the clinical characteristics, clinical cure, recurrence and mortality in patients with CDI treated with either fidaxomicin or vancomycin.

Methods

A retrospective case-control study was conducted on patients with CDI treated with either fidaxomicin or vancomycin at a hospital from January 2019 to March 2022.

Results

We assessed 140 patients with CDI episodes, 70 patients treated with fidaxomicin and 70 with vancomycin. Seventy (50%) were male. Median age was 70 years old (IQR: 56-81). Fidaxomicin group had more recurrent CDI episodes within six months (59% vs 11%, p ≤ 0.001), more severity (43% vs 16%, p ≤ 0.001) and less treatment response (84% vs 100%, p ≤ 0.002) compared with vancomycin group. Recurrence and mortality rates in the follow-up period did not differ in both groups.

Conclusions

Our study found fidaxomicin treatment had worse outcomes due to restricted usage, potentially impacting its effectiveness in CDI. This finding is especially significant for patients with severe or recurrent CDI, as prescribing of the drug was limited until May 2022 in Spain with the lifting of this restriction, further research is necessary to better understand the potential benefits of fidaxomicin in treating CDI.

## Introduction

*Clostridioides difficile* infection (CDI) is a major cause of diarrhea in hospitalized adult patients in both the United States (US) and European countries (EC), entailing a considerable economic and social burden [1,2]. Its prevalence ranged from 10.1 cases per 1000 discharges in the US [3] to 7.3 per 10,000 patient bed days in Europe [4]. Clinical manifestations vary from asymptomatic carriers to mild diarrhea or even death [5]. Many guidelines have identified risk factors associated with CDI recurrence, including patient-related factors such as age, immunosuppression, chronic renal failure, severe underlying medical conditions, prolonged hospital stay, and residency in long-term care facilities, as well as CDI episode-related factors such as concomitant antimicrobials during treatment, previous CDI episodes within the last six months, gastric acid suppression, and severe CDI on presentation onset [2,5,6]. The direct attributable mortality of it has been estimated to be 5%, but this rate may increase up to 30% in recurrence [7]. Indeed, CDI causes higher mortality in elderly patients than their expected mortality, possibly due to unknown factors, even in the absence of recurrent infections [8].

In 2021 CDI guidelines recommend fidaxomicin as the choice treatment for the first episode and first recurrence, as long as it is available [5,6]. This recommendation is based on the main results from pivotal randomized clinical trials (RCT) where fidaxomicin showed higher rates of overall cure and lower rates of recurrence compared to vancomycin [9-11]. These findings have been supported by some observational studies as well [12-14]. However, additional data are necessary to establish the effectiveness and potential benefits of fidaxomicin in specific patient subgroups, including those with severe initial episodes, concomitant antibiotic treatment, and cases of BI/NAP1/027 infections, among others [15,16]. It should be noted that in some of these subgroups, the use of fidaxomicin had not always yielded the expected outcomes. According to the initial recommendation of the European Medicines Agency (EMA) [17], in 2012 Spanish Medicines and Healthcare Product Regulatory Agency (AEMPS) approved fidaxomicin use exclusively for patients in their second or subsequent episodes of recurrence of CDI [18]. Recently, in May 2022, AEMPS approved fidaxomicin for the first episode according to 2021 guidelines [5,19]. This change in Spain allowed us to conduct a study to evaluate and describe clinical characteristics, clinical cure, recurrence rate, and mortality in patients with CDI treated with fidaxomicin compared to vancomycin, in a setting of limited fidaxomicin use.

## Materials and methods

Study design and patients

We conducted a retrospective single-center observational cohort study involving patients older than 16 years old with CDI, comparing the treatment outcomes of fidaxomicin (200 mg every 12 hours orally) and vancomycin (125 mg every 6 hours orally) at La Paz University Hospital from January 2019 to March 2022, with a follow-up period of 24 weeks from the start of CDI therapy. We matched both cohorts based on sex and age. La Paz University Hospital is a 1300-bed teaching hospital with all available specialties and organ transplants. The study was approved by the Ethics Committee for Drug Research (PI-5430).

Objectives

The primary objectives of this study include describing the clinical cure rate after treatment, the percentage of CDI recurrence, and mortality at 12 and 24 weeks post-treatment, while comparing the differences between patients treated with vancomycin and fidaxomicin. Additionally, secondary objectives involve analyzing and identifying risk factors for recurrence, specifying in which episode of ICD fidaxomicin was administered and whether vancomycin had been previously administered, and outlining the percentage of patients who received another concomitant drug along with fidaxomicin.

Definitions and data collection

CDI was defined as positive RT-PCR for *C. difficile* in a stool sample and new-onset unexplained increased stool rate consisting of in ≥3 unformed stools in 24 hours, according to the Infectious Diseases Society of America (IDSA) and the European Society of Clinical Microbiology and Infectious Diseases (ESCMID) criteria [5,6,20]. Only one episode per patient was included in the study period. Patients treated with at least two days of specific anti-CDI treatment and who were older than 16 years of age were included in the study. Patients with incomplete medical records or those who had received less than two days of anti-CDI treatment were excluded. We identified all individuals who received treatment with fidaxomicin for more than two days and determined whether they had experienced an episode of it according to the study's criteria. For vancomycin-treated patients, a 1:1 matching by sex and age was performed with patients who had received treatment for at least two days of vancomycin, considering the same inclusion and exclusion criteria of the study.

Recurrence was defined as the reappearance of CDI symptoms accompanied by a positive reverse transcription polymerase chain reaction (RT-PCR) test for *C. difficile* in stool after resolution of symptoms from a previous episode. Clinical cure was defined as the absence of recurrence during the follow-up period, which included both treatment cure at 48 hours after finishing treatment and sustained cure at 12 weeks after completing treatment. We analyzed recurrence rates at a 12-week follow-up period according to IDSA guidelines [6], and recorded recurrence rates at 4, 8, and 12 weeks [5]. Death was recorded for all study patients during their hospital admission and at 12 and 24 weeks after the initial diagnosis of it. CDI-attributable mortality was considered if no other cause was identified within 10 days of the diagnosis.

We performed a 24-week follow-up since the CDI diagnosis by reviewing the electronic medical records, including possible visits to both primary care centers and hospitals in the Madrid community. For mortality and recurrence, we performed a six-month follow-up. Electronic health records data were used to collect demographics, underlying conditions, risk factors for severe or recurrent CDI, concomitant medications, and outcome variables (clinical cure, recurrence, and death). Data were recorded in a standardized form and anonymized according to the hospital regulations. Immuno-suppression was defined as the use of immunosuppressive therapy (steroid treatment, other immunosuppressor drugs, immunomodulating agents or chemotherapy) and/or the presence of any immunosuppressive illness (cancer, hematological malignancy, HIV infection, organ transplant). Corticosteroid treatment was specifically defined as the use of systemic corticosteroids for more than two weeks or more than 20 mg per day for at least one week in the last two weeks.

Severity, recurrence (first recurrency episode), treatment response, and sustained cure were defined according to ESCMID guidelines (5). Zar score was recorded [21]. Acute renal failure was considered if the creatinine value was greater than 1.5 mg/dL or if the estimated glomerular filtration rate (eGFR) was less than 49 mL/min/1.73m^2^. We recorded the duration of treatment and, we collected data on patients who received extended treatment, either through vancomycin tapering or extended fidaxomicin courses.

Statistical analysis

The median and IQR were used for quantitative variables and absolute and relative frequencies for qualitative variables. To compare the two groups, we used the Mann-Whitney U test for quantitative variables (due to their non-normality) and the Chi-square test (or Fisher's exact test) for qualitative variables. For variables with three or more categories, we applied Holm's post-hoc test. Survival curves were generated using the Kaplan-Meier method and compared with the log-rank test. Statistical significance was defined as a p-value ≤ 0.05. Statistical analyses were conducted using R software (version 4.1.1, R Core Team (2020), Vienna, Austria).

## Results

Patient population and baseline characteristics

**Figure 1:**
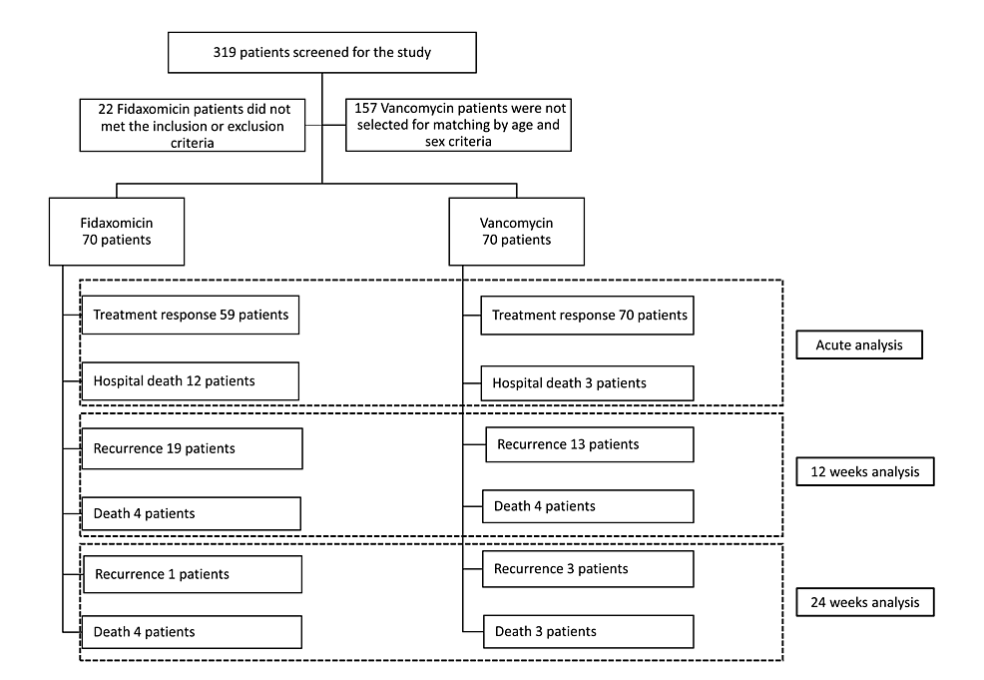
Patients flow of the study

During the study period, we included a total of 70 patients with CDI who met the inclusion criteria and none of the exclusion criteria. We also included 70 patients who were treated with vancomycin and matched for sex and age, and who met the criteria for the study. The flowchart in Figure 1 provides an overview of the patient selection process. Fifty percent were male, with a median age of 70 years (IQR: 56-81).

At baseline, patients treated with fidaxomicin had a higher incidence of severe cases (43% vs. 16%, p ≤ 0.001), a higher Zar score (33% vs. 9% with Zar Score >2, p ≤ 0.001), and a higher number of CDI previous recurrences at inclusion (59% vs. 11%, p ≤ 0.001) than those treated with vancomycin. Twenty-seven patients received fidaxomicin in their second episode and 14 in their third episode, while only eight received vancomycin in the second episode (Table 1).

**Table 1:** Baseline patients and clinical characteristics * Statistically significant variable (p-value <0.05) PPIs: Proton pump inhibitors, CDI: *Clostridioides difficile* infection, ESCMID: European Society of Clinical Microbiology and Infectious Diseases, ICD: International Classification of Diseases

	Overall N = 140 (%)	Fidaxomicin N = 70 (%)	Vancomycin N = 70 (%)	p-value
Baseline characteristics (%)				
Male	70 (50)	35.0 (50)	35.0 (50)	1.00
Age (median [IQR])	70 (56, 81)	70 (56, 81)	70 (56, 81)	0.99
Inpatient (%)	101 (72)	49 (70)	52 (74)	0.71
Immunocompromised (%)	62 (44)	29 (41)	33 (47)	0.61
PPIs (%)	71 (51)	35 (50)	36 (51)	1.00
Renal failure (%)	29 (21)	18 (26)	11 (16)	0.21
Concomitant antibiotic (%)	58 (42)	30 (43)	28 (41)	0.92
CDI 6 months prior (%)	49 (35)	40 (59)	6 (9)	<0.01*
Clinical features				
Severe CDI 2021 ESCMID criteria (%)	41 (29)	30 (43)	11 (16)	0.001*
ICD Fulminant (%)	7 (5)	7 (10)	0 (0)	0.02*
Zar’s score ≥2 (%)	29 (21)	23 (33)	6 (9)	0.01*
ICU admission (%)	3 (2)	3 (4)	0 (0)	0.24
CDI status for treatment (%)				<0.01*
Primary episode	91 (65)	29 (41)	62 (89)	
1 previous CDI	35 (25)	27 (39)	8 (11)	
>1 previous CDI	14 (10)	14 (20)	0 (0)	
Treatment				
Duration of therapy (days), median [IQR]	10 [10, 10]	10 [10, 10]	10 [10, 10]	0.42
Tappering (%)	6 (4)	5 (7)	1 (1)	0.21
Bezlotoxumab associated (%)	20 (14)	14 (20)	6 (9)	0.09

CDI episode treatment

Both groups received treatment for a median of 10 days [IQR: 10-10]. Five patients (7%) in the fidaxomicin group received extended-pulse or tapering therapy, compared to only one patient (1%) in the vancomycin (Table 1). Concomitant anti-CDI treatment was used in 32 patients in the fidaxomicin group and 13 patients in the vancomycin group. The most frequently used therapies were bezlotoxumab, oral vancomycin, and tigecycline in 14, six, and seven patients, respectively, in the fidaxomicin group, and Bezlotoxumab and intravenous metronidazole in six and seven patients, respectively, in the vancomycin group.

Outcome variables

Globally, 92% (129) of patients achieved a treatment response. All patients in the vancomycin group responded to treatment at the end of therapy, but only 84% (59) in the fidaxomicin group did (p ≤0.002). Although treatment response rates for high-risk patients (age ≥65 years, receiving concomitant antibiotic therapy, previous six-month episode of CDI) did not differ significantly between groups, the results were better in the vancomycin group (Tables 2, 3).

**Table 2:** Outcome of the study Statistically significant variable: p-value <0.05 Number of patients in the time point @At start of study: overall = 140, fidaxomicin group = 70, vancomycin group = 70 #At 12 weeks: overall = 125, fidaxomicin group = 58, vancomycin group = 67 (excluded deaths during admissions) $At 24 weeks: overall= 117, fidaxomicin group = 54, vancomycin group = 63 (excluded all deaths previous 12 weeks)

	Overall	Fidaxomicin	Vancomycin	p-value
Clinical cure (%)				
Treatment response, n = 140 (%) ^@^	129 (92)	59 (84)	70 (100)	<0.01*
Sustained cure, n = 125 (%) ^#^	89 (64)	36 (62)	53 (79)	0.05*
Recurrence				
Recurrence at 12 weeks (%), 125 (%) ^#^	31 (25)	19 (33)	18 (19)	0.60
Recurrence between 12-24 weeks (%), 117 (%)^ $^	4 (3)	1 (2)	3 (5)	0.72
Death (%)				
During admission	15 (11)	12 (17)	3(4)	0.01*
At 12 weeks ^#^	23 (18)	16 (28)	7 (10)	0.07
At 24 weeks ^$^	7 (6)	4 (7)	3 (5)	0.77
Attributable death	5 (4)	4 (6)	1 (1)	0.91

**Table 3:** Subgroup outcomes of treatment response, sustained cure, and risk factors CDI in the study analysis populations Number of patients in the time point &At 4 weeks: overall = 140, fidaxomicin group = 70, vancomycin group = 70 %At 8 weeks: overall = 129, fidaxomicin group = 61, vancomycin group = 68 #At 12 weeks: overall = 125, fidaxomicin group = 58, vancomycin group = 67 $At 24 weeks: overall= 117, fidaxomicin group = 54, vancomycin group = 63 (excluded all deaths previous 12 weeks) CDI: *Clostridioides difficile* infection, ESCMID: European Society of Clinical Microbiology and Infectious Diseases, FIX: fidaxomicin, VAN: vancomycin

Outcome, n (%)	FIX	VAN	Age				Concomitant antibiotics				Number of CDI recurrences at inclusion						Severe CDI 2021 ESCMID criteria (%)			
			FIX		VAN		FIX		VAN		Primary episode		1 previous CDI		≥1 previous CDI		Severe		Non-severe	
			≥65 y	<65 y	≥65 y	<65 y	Yes 30	No 40	Yes 28	No 41	FIX 29	VAN 62	FIX 27	VAN 8	FIX 14	VAN 0	FIX 30	VAN 11	FIX 40	VAN 59
Treatment response N= 129/140	59/70 (84)	70/70 (100)	38/45 (84)	21/25 (84)	45/45 (100)	25/25 (100)	23/30 (77)	36/40 (90)	28/28 (100)	41/41 (100)	22/29 (76)	62/62 (100)	25/27 (93)	8/8 (100)	12/14 (86)	0/0 (0)	22/30 (73)	11/11 (100)	36/40 (90)	59/59 (100)
Sustained clinical cure N= 89/125 ^#^	36/58 (62)	53/67 (79)	23/37 (62)	13/21 (62)	34/43 (79)	19/24 (79)	15/20 (75)	21/38 (55)	23/26 (89)	29/53 (55)	13/20 (65)	47/59 (80)	15/24 (63)	6/8 (75)	8/14 (57)	0/0 (0)	12/21 (57)	9/10 (82)	24/37 (65)	44/57 (77)

Sustained cure was achieved in 71% (89/125) of the patients, with 62% (36/58) in the fidaxomicin group and 79% (53/67) in the vancomycin group. Further analysis by factors such as age, concomitant antibiotics, number of CDI recurrences at inclusion, and criteria for severe showed no significant differences between the two groups (see Tables 2, 3 for details).

At 12 weeks of follow-up, CDI recurred in 26% (32/125) patients and at 24 weeks in 4% (4/117), with no difference between treatment groups (Tables 2, 4). CDI recurrence episodes appeared in 10% (14/140) within the first month, 9% (11/129) within the second month, 5% (6/125) within the third month, and 4% (4/117) between three and six months (Table 3). At 24 weeks, the recurrence rate was 2% (1/54) in the fidaxomicin group and 5% (3/63) in the vancomycin group.

**Table 4:** Number of recurrences according to time Number of patients in the time point &At 4 weeks: overall = 140, fidaxomicin group = 70, vancomycin group = 70 %At 8 weeks: overall = 129, fidaxomicin group = 61, vancomycin group = 68 #At 12 weeks: overall = 125, fidaxomicin group = 58, vancomycin group = 67 $At 24 weeks: overall= 117, fidaxomicin group = 54, vancomycin group = 63 (excluded all deaths previous 12 weeks) FIX: fidaxomicin, VAN: vancomycin

Recurrence, n (%)	FIX	VAN
			<4 weeks^&^ N= 14/140	8/70 (11)	7/70 (10)
			<8 weeks^%^ N=11/129	7/61 (12)	4/68 (6)
<12 weeks^#^ N=6/125	4/58 (5)	2/67 (3)
12-24 weeks^$^ N=4/117	1/54 (2)	3/63 (5)

**Figure 2:**
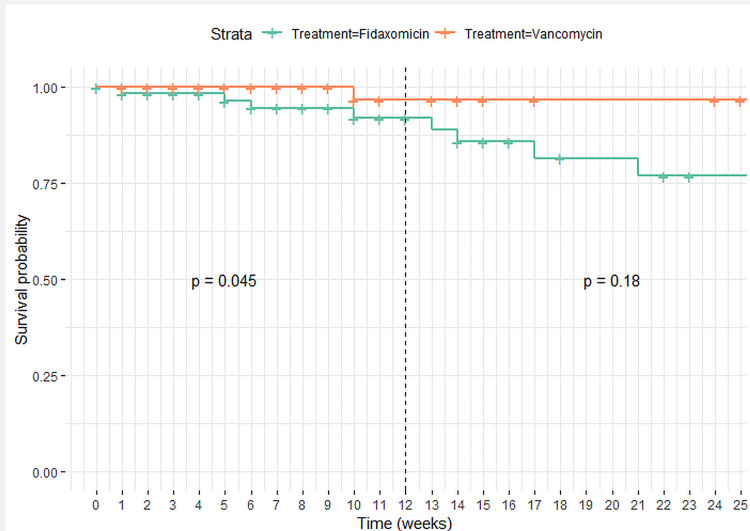
Survival curves

Kaplan-Meier survival analysis showed that patients treated with oral vancomycin had a greater survival rate than those treated with oral fidaxomicin at 12 weeks (p = 0.045) (Figure 2). However, univariate analysis at defined time points did not show this difference except for in-hospital mortality (Table 2 and Figure 2). Attributable CDI deaths were four in the fidaxomicin group and one in the vancomycin group.

## Discussion

In this retrospective case-control study, we described the effectiveness of fidaxomicin in the treatment of CDI in a single center from January 2019 to March 2022 during a period of limited fidaxomicin use by the AEMPS. Our findings revealed that fidaxomicin was prescribed for second or subsequent episodes of CDI and/or for patients with more severe cases in 58% of patients, compared to only 11% in the vancomycin group (which was limited to first-recurrence cases only). This difference was statistically significant (p≤ 0.01) and based on current recommendations.

Patients who received vancomycin had higher clinical cure rates compared to those who received fidaxomicin, and we observed statistically significant differences in both treatment response rate and sustained cure. Furthermore, patients treated with fidaxomicin had a higher 12-week mortality rate than those treated with vancomycin, indicating worse outcomes for the former group. Moreover, we observed no significant differences in CDI recurrence rates between the two treatments. Taken together, these results suggest that vancomycin may be a more effective treatment option for CDI in the context of limited use when compared to fidaxomicin. New drugs for treating and preventing the recurrence of CDI, such as fidaxomicin since 2011 [9,10] and bezlotoxumab since 2016 [22], have been available, but their adoption into clinical practice has been slower than expected. The delayed implementation of fidaxomicin in clinical practice may be attributed to the limited usage recommended by AEMPS [18], EMA [17] or health insurance in the US [23] probably to an economic component.

Fidaxomicin pivotal RCTs, which included mainly first CDI episodes, showed a reduction in recurrence rates (13% of recurrence in the fidaxomicin group) when compared to vancomycin with similar sustained clinical cure rates (92%) in both groups at 30 days [9,10]. These results were reproduced in smaller clinical trials [24,25]. However, several real-life observational studies [12,16,26] showed that fidaxomicin was used for second or subsequent CDI episodes, as we found in our study. Another study [14] with a higher percentage of patients treated in the first episode replicated the results of the pivotal trials. Nevertheless, all these post-authorization studies [12,14,26,27] confirmed the outcomes in both clinical cure and recurrence rate that appeared in the RCT [9,10] varying from 82% to 92% cure [12,14,26,27], with recurrence rate from 15% to 28% [12,14,16,26,28], similar to our results (cure 84% and recurrence 33% in fidaxomicin group). In addition, Gentry et al. reported higher failure rates in patients with severe CDI treated with fidaxomicin compared to vancomycin (9.39% vs. 1.41%), as well as higher combined outcome clinical failure or recurrence at 12 weeks (32% in fidaxomicin vs 25% in vancomycin) [16]. Neither, in Tieu et al.'s study, which compared the use of fidaxomicin or vancomycin for treating the first or second recurrent CDI episode, found no significant differences between the two treatments in the combined outcome of clinical failure or recurrence at 90 days [28]. In our series, 40% of patients treated with fidaxomicin had a severe episode of CDI, a rate similar to those reported by Escudero et al. [12] or Guery et al. [14], but higher than Giacobbe et al.'s (28%) [27]. Probably, in our series, the better outcomes observed in vancomycin-treated patients compared to those treated with fidaxomicin were due, in part, to the fact that the vancomycin group had less severe disease and higher first episodes. While Guery et al.'s study [14] was similar in design to ours, their results differed, likely due to a higher proportion of first episodes in their fidaxomicin group (63% vs. 41% in ours). A recent meta-analysis of fidaxomicin RCTs concluded that fidaxomicin was associated with significantly higher overall cure rates and significantly lower recurrence rates than vancomycin, but the recommendations for its use were strong for non-severe cases and first episodes [15,16].

Our study has a long-term follow-up period of six months, allowing us to analyze mortality in the short, medium, and long term. Overall, we observed a higher mortality rate in the fidaxomicin group (20 out of 70) compared to the vancomycin group (10 out of 70). In addition, the mortality attributed to CDI was also higher in the fidaxomicin group, with four patients compared to one patient in the vancomycin group. The majority of studies on fidaxomicin have not assessed CDI-associated mortality. In the DAFNE study, 12-week mortality was 12%, with a CDI-attributable mortality of 1.3% (3 patients) [14]. In another study, 11.8% died at 12 weeks among those treated with fidaxomicin [12]. In a report of long-term mortality analysis of patients with severe CDI, no significant differences in mortality were observed between the two treatment groups at day 30, day 90 and day 180 [16]. Recent cost and mortality assessment studies in CDI showed a 30-day mortality rate of 15% and 20.4% at one year, with an at-attributable mortality of 4.3% [29]. This mortality was found to be higher for recurrent episodes compared to first episodes, and which was observed both in the short term and long term [7]. It is worth noting that the excess mortality associated with CDI was not limited to the short term, as it was also observed a year later for unknown reasons [8].

Study’s limitations

Our research targeted the efficacy and safety of fidaxomicin in real-life CDI treatment. Outcomes with fidaxomicin, diverging from clinical trials, could stem from different patient populations. It might harbor biases in study group selection, focusing on age and gender, without accounting for the specific treatment episode. Notably, fidaxomicin was prescribed more to patients at greater therapeutic failure risk. Our results shed light on fidaxomicin's real-world efficacy but generalizing them requires caution. We couldn't conduct antibiotic susceptibility studies due to the study's retrospective nature and our center's limitations. Additionally, our microbiology laboratory does not monitor for emerging resistance, given that the relationship between minimum inhibitory concentrations (MICs) and CDI clinical outcomes is not well-established.

## Conclusions

Our results suggest that the lack of superiority of fidaxomicin over vancomycin in terms of clinical cure may be attributed to the differences in patient severity between the two groups. Fidaxomicin was predominantly used in patients with more severe episodes and/or a higher risk of recurrence. Our findings support current guidelines that recommend the use of fidaxomicin for CDI first episodes, while reserving vancomycin treatment for recurrences or more severe episodes. However, further studies are needed to confirm these outcomes.
